# Supplementary material for: The Role of Iron-Chelating Therapy in Improving Neurological Outcome in Patients with Intracerebral Hemorrhage: Evidence-Based Case Report
Source: Medicina (Kaunas). 2023 Feb 24;59(3):453. doi: 10.3390/medicina59030453 (PMC10058021; doi:10.3390/medicina59030453)
Supplement: Supplementary file 1 [file medicina-59-00453-s001.zip › medicina-2170329-supplementary.pdf]

Supplementary Table S1. Terminology used for literature search

Database	Search strategy	Hits	Selected Articles
EMBASE	(('brain hemorrhage'/exp OR 'bleeding, corpus callosum' OR 'brain bleeding' OR 'brain haemorrhage' OR 'brain hemorrhage' OR 'brain microhaemorrhage' OR 'brain microhemorrhage' OR 'cerebral haemorrhage' OR 'cerebral hemorrhage' OR 'cerebral microbleed' OR 'corpus callosum bleeding' OR 'corpus callosum haemorrhage' OR 'corpus callosum hemorrhage' OR 'encephalorrhagia' OR 'haemorrhage, brain' OR 'haemorrhage, intracranial' OR 'haemorrhagic apoplexy' OR 'haemorrhagic stroke' OR 'haemorrhagic stroke intracerebral bleeding' OR 'hematencephalon' OR 'hemorrhage, brain' OR 'hemorrhage, intracranial' OR 'hemorrhagic apoplexy' OR 'hemorrhagic stroke' OR 'hemorrhagic stroke intracerebral bleeding' OR 'hypertensive intracranial haemorrhage' OR 'hypertensive intracranial hemorrhage' OR 'intracerebral bleeding' OR 'intracerebral haemorrhage' OR 'intracerebral hemorrhage' OR 'intracortical haemorrhage' OR 'intracortical hemorrhage' OR 'intracranial bleeding' OR 'intracranial haemorrhage' OR 'intracranial haemorrhage, hypertensive' OR 'intracranial haemorrhages' OR 'intracranial hemorrhage' OR 'intracranial hemorrhage, hypertensive' OR 'intracranial hemorrhages' OR 'intraventricular haemorrhage' OR 'intraventricular hemorrhage' OR 'periventricular haemorrhage' OR 'periventricular hemorrhage' OR 'posterior fossa haemorrhage' OR 'posterior fossa hemorrhage') OR ('subarachnoid hemorrhage'/exp OR 'aneurysmal subarachnoid haemorrhage' OR 'aneurysmal subarachnoid hemorrhage' OR 'arachnoid haemorrhage, brain' OR 'arachnoid hemorrhage, brain' OR 'arachnoidal bleeding' OR 'arachnoidal haemorrhage' OR 'arachnoidal haemorrhage, brain' OR 'arachnoidal hemorrhage' OR 'arachnoidal hemorrhage, brain' OR 'bleeding, subarachnoid' OR 'brain arachnoid haemorrhage' OR 'brain arachnoid hemorrhage' OR 'haemorrhage, subarachnoid' OR 'hemorrhage, subarachnoid' OR 'spontaneous subarachnoid haemorrhage' OR 'spontaneous subarachnoid hemorrhage' OR 'subarachnoid bleeding' OR 'subarachnoid blood' OR 'subarachnoid haematoma' OR 'subarachnoid haemorrhage' OR 'subarachnoid haemorrhage, brain' OR 'subarachnoid hematoma' OR 'subarachnoid hemorrhage' OR 'subarachnoid hemorrhage, brain' OR 'subarachnoid hemorrhagia' OR 'subarachnoidal bleeding' OR 'subarachnoidal haemorrhage' OR 'subarachnoidal hemorrhage') OR ('subdural hematoma'/exp OR 'acute subdural haematoma' OR 'acute subdural hematoma' OR 'chronic subdural haematoma' OR 'chronic subdural haematomata' OR 'chronic subdural hematoma' OR 'chronic subdural hematomata' OR 'haematoma, subdural' OR 'haematoma, subdural, acute' OR 'haematoma, subdural, chronic' OR 'haematoma, subdural, intracranial' OR 'haemorrhage, subdural' OR 'haemorrhagic pachymeningitis' OR 'hematoma, subdural' OR 'hematoma, subdural, acute' OR 'hematoma, subdural, chronic' OR 'hematoma, subdural, intracranial' OR 'hemorrhage, subdural' OR 'hemorrhagic pachymeningitis' OR 'intracranial subdural haematoma' OR 'intracranial subdural haematomas' OR 'intracranial subdural haematomata' OR 'intracranial subdural hematoma' OR 'intracranial subdural hematomas' OR 'intracranial subdural hematomata' OR 'pachymeningiosis haemorrhagica interna' OR 'pachymeningitis haemorrhagica' OR 'subdural bleeding' OR 'subdural haematoma' OR 'subdural haemorrhage' OR 'subdural hematoma' OR 'subdural hemorrhage' OR	35	8

	'subepidural haematoma' OR 'subepidural hematoma')) AND (('iron chelation'/exp OR 'chelation, iron' OR 'fe chelation' OR 'iron chelation') OR ('iron chelating agent'/exp OR 'iron chelating agent' OR 'iron chelating agents' OR 'iron chelating compound' OR 'iron chelating drug' OR 'iron chelator' OR 'iron chelators') OR (deferoxamine/exp OR 'ba 29837' OR 'ba29837' OR 'deferoxamine' OR 'deferoxamine b' OR 'deferriferrioxamine b' OR 'deferrioxamine' OR 'deferrioxamine b' OR 'desferoxamine' OR 'desferrioxamine' OR 'desferrioxamine b' OR 'nsc 527604') OR ('deferoxamine mesylate'/exp OR 'ba 33112' OR 'ba33112' OR 'cgh 749b' OR 'cgh749b' OR 'deferoxamine mesilate' OR 'deferoxamine mesylate' OR 'deferoxamine methanesulfonate' OR 'deferrioxamine mesylate' OR 'desferal' OR 'desferin' OR 'desferol' OR 'desferrioxamine b mesylate' OR 'desferrioxamine mesylate' OR 'desferrioxamine methanesulfonate' OR 'dfom' OR 'icl 749b' OR 'icl749b') OR (deferasirox/exp OR '4 [3, 5 bis (2 hydroxyphenyl) 1, 2, 4 triazol 1 yl] benzoic acid' OR '4 [3, 5 bis (2 hydroxyphenyl) 1h 1, 2, 4 triazol 1 yl] benzoic acid' OR 'cgp 72670' OR 'cgp72670' OR 'deferasirox' OR 'desirox' OR 'dst 0509' OR 'dst0509' OR 'exjade' OR 'icl 670' OR 'icl 670a' OR 'icl670' OR 'icl670a' OR 'jadenu' OR 'jadenu sprinkle') OR (deferiprone/exp OR '1, 2 dimethyl 3 hydroxy 4 pyridone' OR '1, 2 dimethyl 3 hydroxypyrid 4 one' OR '1, 2 dimethyl 3 hydroxypyridin 4 one' OR '3 hydroxy 1, 2 dimethyl 1, 4 dihydro 4 pyridinone' OR '3 hydroxy 1, 2 dimethyl 1, 4 dihydropyridin 4 one' OR '3 hydroxy 1, 2 dimethyl 4 pyridinone' OR '3 hydroxy 1, 2 dimethyl 4 pyridone' OR '3 hydroxy 1, 2 dimethylpyrid 4 one' OR 'apo 066' OR 'apo 66' OR 'apo066' OR 'apo66' OR 'cgp 37391' OR 'cgp37391' OR 'cp 020' OR 'cp 20' OR 'cp020' OR 'cp20' OR 'crmd 001' OR 'crmd001' OR 'deferiprone' OR 'deferrum' OR 'deferum' OR 'ferriprox' OR 'kelfer' OR 'l 1' OR 'upkanz')) AND (('National Institutes of Health Stroke Scale'/exp OR 'NIH Stroke Scale' OR 'NIH Stroke Score' OR 'NIHSS' OR 'National Institute of Health Stroke Scale' OR 'National Institute of Health Stroke Score' OR 'National Institutes of Health Stroke Scale' OR 'National Institutes of Health Stroke Score') OR ('Rankin scale'/exp OR 'Rankin scale' OR 'modified Rankin scale') OR ('Barthel index'/exp OR 'Barthel ADL index' OR 'Barthel index'))		
Cochrane	[("hemorrhagic stroke"):ti,ab,kw OR ("intracerebral haematoma"):ti,ab,kw OR ("intracerebral haemorrhage"):ti,ab,kw OR ("intracranial hematoma"):ti,ab,kw OR ("intracranial hemorrhage"):ti,ab,kw OR ("subarachnoid haemorrhage"):ti,ab,kw OR ("epidural hematoma"):ti,ab,kw OR ("subdural hematoma"):ti,ab,kw OR ("subdural hemorrhage"):ti,ab,kw OR MeSH descriptor: [Cerebral Hemorrhage] explode all trees MeSH descriptor: [Hemorrhagic Stroke] explode all trees] AND [MeSH descriptor: [Iron Chelating Agents] explode all trees OR MeSH descriptor: [Deferoxamine] explode all trees OR MeSH descriptor: [Deferiprone] explode all trees OR MeSH descriptor: [Deferasirox] explode all trees OR (iron chelating agents):ti,ab,kw OR (deferasirox):ti,ab,kw OR (deferiprone):ti,ab,kw OR ("deferoxamine mesylate"):ti,ab,kw OR (deferoxamine):ti,ab,kw] AND [(NIHSS):ti,ab,kw OR (National Institutes of Health Stroke Scale):ti,ab,kw OR (modified rankin scale):ti,ab,kw OR (mRS):ti,ab,kw OR (outcome):ti,ab,kw OR (barthel index):ti,ab,kw OR (barthel scale):ti,ab,kw OR (neurological outcome):ti,ab,kw]	8	5
Pubmed	**(((((((((((((((((((cerebral hemorrhage[MeSH Terms]) OR (intracranial hemorrhage[MeSH Terms])) OR (intracranial hemorrhag*[Title/Abstract])) OR (intracranial haemorrhag*[Title/Abstract])) OR (intracerebral**	8	5

	**hemorrhag*[Title/Abstract])) OR (intracerebral haemorrhag*[Title/Abstract])) OR (intracerebral hematoma[Title/Abstract])) OR (intracerebral haematoma[Title/Abstract])) OR (subarachnoid hemorrhag*[Title/Abstract])) OR (subarachnoid haemorrhag*[Title/Abstract])) OR (SAH[Title/Abstract])) OR (subdural hemorrhag*[Title/Abstract])) OR (subdural haemorrhag*[Title/Abstract])) OR (epidural haemorrhag*[Title/Abstract])) OR (epidural hemorrhag*[Title/Abstract])) OR (intraventricular hemorrhag*[Title/Abstract])) OR (intraventricular haemorrhag*[Title/Abstract])) OR (IVH[Title/Abstract])) OR (hemorrhagic stroke[Title/Abstract])) OR (haemorrhagic stroke[Title/Abstract])) AND ((((((((((iron chelating agents[MeSH Terms]) OR (deferoxamine[MeSH Terms])) OR (deferiprone[MeSH Terms])) OR (deferasirox[MeSH Terms])) OR (iron chelation[Title/Abstract])) OR (iron chelating agent[Title/Abstract])) OR (deferoxamine[Title/Abstract])) OR (desferrioxamine[Title/Abstract])) OR (deferasirox[Title/Abstract])) OR (deferiprone[Title/Abstract]))) AND ((((((((National Institutes of Health Stroke Scale[Title/Abstract]) OR (NIHSS[Title/Abstract])) OR (modified rankin scale[Title/Abstract])) OR (modified rankin scales[Title/Abstract])) OR (outcom*[Title/Abstract])) OR (neurological defici*[Title/Abstract])) OR (barthel index[Title/Abstract])) OR (barthel scale[Title/Abstract]))**		
Google Scholar	“Intracerebral Hemorrhage” OR “Intracranial Hemorrhage” OR “Subarachnoid Hemorrhage” OR “Subdural Hemorrhage” OR “Intraventricular Hemorrhage” OR “Hemorrhagic Stroke” AND “Iron Chelating” OR “Iron Chelation” OR Deferoxamine OR Desferrioxamine OR Deferiprone OR Deferasirox AND “Neurological Outcome” OR “National Institutes of Health Stroke Scale” OR NIHSS OR “modified Rankin Scale” OR mRS	67	7
